# Emerging concepts in osteoarthritis and musculoskeletal diseases: Insights from the University of Debrecen Musculoskeletal Symposium 2025

**DOI:** 10.1016/j.ocarto.2025.100728

**Published:** 2025-12-11

**Authors:** Patrik Kovács, Judit Vágó, Ali Mobasheri, Zsuzsa Jenei-Lanzl, Frank Zaucke, Henning Madry, László Csernoch, Zoltán Szekanecz, Zsuzsa Szondy, Árpád Szöőr, Istvan Szatmari, Tamás Oláh, Csaba Matta

**Affiliations:** a Department of Anatomy, Histology and Embryology, Faculty of Medicine, University of Debrecen, Debrecen, Hungary; b Research Unit of Health Sciences and Technology, Faculty of Medicine, University of Oulu, Oulu, Finland; c Department of Regenerative Medicine, State Research Institute Centre for Innovative Medicine, Vilnius, Lithuania; d Department of Joint Surgery, First Affiliated Hospital of Sun Yat-sen University, Guangzhou, China; e Faculty of Medicine, University of Liège, Liège, Belgium; f Dr. Rolf M. Schwiete Research Unit for Osteoarthritis, Department of Trauma Surgery and Orthopedics, Goethe University Frankfurt, University Hospital, Frankfurt am Main, Germany; g Center of Experimental Orthopaedics, Saarland University, Homburg, Germany; h Department Physiology, Faculty of Medicine, University of Debrecen, Debrecen, Hungary; i Department of Rheumatology, Faculty of Medicine, University of Debrecen, Debrecen, Hungary; j Division of Dental Biochemistry, Department of Basic Medical Sciences, Faculty of Dentistry, University of Debrecen, Debrecen, Hungary; k Department of Biochemistry and Molecular Biology, Faculty of Medicine, University of Debrecen, Debrecen, Hungary; l Department of Biophysics and Cell Biology, Faculty of Medicine, University of Debrecen, Debrecen, Hungary

**Keywords:** Osteoarthritis, Musculoskeletal disorders, Intervertebral disc, Cartilage, Muscle regeneration, Symposium

## Abstract

**Objective:**

To highlight the key translational advances and interdisciplinary discussions from the 2025 University of Debrecen Musculoskeletal Symposium (UD-MUSK), focusing on osteoarthritis (OA) and related musculoskeletal (MSK) diseases.

**Method:**

The UD-MUSK Symposium convened international and local experts for keynote presentations and scientific dialogue spanning OA and MSK disease mechanisms, translational therapies, and tissue regeneration. Core topics included extracellular matrix (ECM) remodelling, cell/gene therapies, animal disease models, chronic stress, autoimmunity, immunotherapies, and muscle and stem cell biology, reflecting the Symposium's broad interdisciplinary spectrum.

**Results:**

ECM proteomics revealed degradation fragments that act as drivers and potential modulators of OA, offering new biomarkers and therapeutic targets. Advances in cell- and gene-based therapies were presented, including the use of growth differentiation factor 6 (GDF6) and CRISPR approaches for cartilage and intervertebral disc repair. Large animal models simulating spatio-temporal changes in human OA, and studies demonstrating chronic stress-accelerated OA via neuroendocrine mechanisms, were highlighted. Novel strategies in immune modulation, such as chimeric antigen receptor (CAR) T cell therapy for autoimmune myopathies, alongside research on muscle regeneration and circadian regulation by mechanical loading, further exemplified the translational breadth of the meeting.

**Conclusion:**

The symposium underscored the value and need of integrating molecular research and disease modeling. Interdisciplinary collaboration continues to enhance understanding of MSK disease mechanisms and supports development of targeted, regenerative therapies with translational potential for OA and related disorders.

## 1 Introduction

The University of Debrecen Musculoskeletal Symposium (UD-MUSK), which took place in Debrecen, Hungary, in May 2025, marked a major milestone in advancing the field of musculoskeletal (MSK) research in Hungary, with a particular focus on osteoarthritis (OA), intervertebral disk degeneration, and muscle-related conditions. This interdisciplinary event convened leading scientists and clinicians, including international experts from prominent European research centres, fostering collaborative exchange. The symposium reinforced the role of the University of Debrecen as a leader in MSK research in Central and Eastern Europe, and its commitment to addressing pressing challenges in MSK disease diagnosis, prevention, and treatment.

MSK disorders, encompassing OA, rheumatoid arthritis (RA), IVDD, and sarcopenia, continue to impose a serious global health burden [1,2]. These complex diseases arise from multifactorial causes, including tissue degeneration, aging populations, comorbidities, environmental factors, sedentary lifestyles, and dietary habits [3,4]. These conditions increasingly affect younger individuals, highlighting biological and social trends [5]. Despite advances in clinical management improving patient quality of life, existing therapies predominantly offer symptom relief without addressing the underlying pathomechanisms [6].

The UD-MUSK symposium provided a platform for keynote presentations by renowned international and local experts, covering disease biomarkers, animal models, autoimmune pathologies, muscle regeneration, and stem cell differentiation. This forum promoted interdisciplinary dialogue between basic science and clinical practice, encouraging new scientific collaborations aimed at innovative therapeutic targets and advanced, locally acting regenerative strategies with fewer systemic effects.

Speakers included distinguished researchers who presented key insights into emerging diagnostic biomarkers, novel disease models, and regenerative therapies: Prof. Ali Mobasheri (University of Oulu, Finland), a leading expert in OA and in IVD degeneration; PD Dr. Zsuzsa Jenei-Lanzl (Goethe University Frankfurt, Germany), focused on the interplay between chronic stress and OA pathogenesis; Prof. Frank Zaucke (Goethe University Frankfurt), specializing in extracellular matrix (ECM) biology in skeletal diseases; and Prof. Henning Madry (Saarland University, Germany), a clinician-scientist advancing spatio-temporal modelling of OA. The program also featured short talks by University of Debrecen researchers Prof. László Csernoch on cytoskeletal roles in muscle regeneration, Prof. Zoltán Szekanecz on autoimmunity and autoinflammation, Prof. Zsuzsa Szondy on adenosine receptor functions in muscle repair, Árpád Szőőr on chimeric antigen receptor T cell innovations for MSK diseases, István Szatmári on RUNX3-driven mesodermal differentiation, and Csaba Matta on mechanotransduction pathways in differentiating chondrocytes. Together, these distinguished speakers presented novel insights into biomarkers, disease models, and regenerative therapies, emphasizing interdisciplinary collaboration essential for clinical translation and advancing precision medicine in musculoskeletal disorders.

**Fig. 1:**
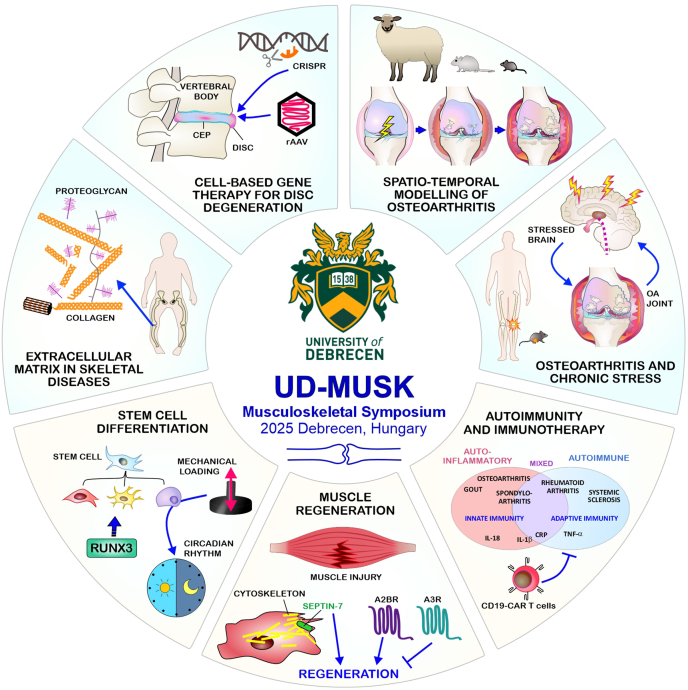
Emerging concepts in osteoarthritis (OA) and musculoskeletal (MSK) diseases presented at the UD-MUSK 2025. The central theme illustrates the symposium's focus on interdisciplinary progress across nine major research domains: ECM remodelling in skeletal disease, cell-based gene therapy for intervertebral disc degeneration, spatio-temporal animal models of OA, the interplay of chronic stress and joint pathology, autoimmunity and innovative immunotherapeutic strategies (including CAR-T cell therapy), mechanisms and targets for muscle regeneration (cytoskeletal and adenosine receptor pathways), as well as stem cell differentiation driven by gene regulatory networks and mechanotransduction. The figure integrates these nine domains, such as the identification of ECM fragments as biomarkers and therapeutic targets, the development of novel cell-based and gene therapies, and the application of disease modelling to enhance clinical translation. It also depicts challenges and future directions: bridging basic science and clinical practice, emphasizing early intervention and regenerative strategies, and fostering collaboration to overcome barriers in diagnostic and therapeutic innovation. The figure encapsulates the Symposium's key message in advancing precision medicine and holistic management for OA and related MSK disorders.

Consensus was reached on the importance of interdisciplinary collaboration as essential for clinical translation. Future directions identified encompassed refining targeted therapies, enhancing biomarker validation, and integrating multi-omics approaches to advance precision medicine in MSK diseases. The following sections of this paper provide a synthesis of the Symposium's major lectures and talks, illustrating both the dynamism and depth of current MSK research at the University of Debrecen and its international partners (Fig. 1).

## 2 The extracellular matrix in common and rare skeletal diseases

Skeletal tissues are characterized by low cellularity and abundant ECM. Traditionally viewed as a structural scaffold providing mechanical support and compartment barriers, recent studies reveal that ECM also sequesters and stores growth factors is critically involved in mechanotransduction, enabling cells to sense mechanical signals crucial for tissue homeostasis [7].

Proteomic analyses show cartilage and bone ECM comprise several hundreds of components, including collagens, proteoglycans, glycoproteins, and non-glycosylated proteins [8]. Data from genetically modified mouse models lacking specific ECM components [9], and monogenetic human diseases, suggest ECM is central, and often causative, in many MSK diseases [10] ranging from rare osteochondrodysplasias to degenerative conditions like OA [11].

In OA, cartilage ECM degradation is driven by numerous proteases whose activity not only leads to tissue loss but also generates matrix fragments used as disease biomarkers [12]. Specific fragments trigger inflammation and protease activation [13], provoke autoantibody production [14], or activate Toll-like receptors such as a 32-mer aggrecan fragment that induces CCL2 and drives OA pain via TLR2 [15]. While most fragments accelerate degeneration, recent studies highlight several with pro-regenerative potential [16].

Current research in the Zaucke group is focusing on systematically analysing ECM degradation in degenerative diseases [17]. Using recombinant ECM components, proteases, and diverse cellular, tissue and animal models, they investigate the role of specific ECM fragments in disease progression. Their goal is to identify not only novel biomarkers but also druggable targets and pro-regenerative ECM fragments for cartilage restoration and innovative OA therapies [18].

Recent studies of the group expand understanding of dynamic ECM remodelling in muscle and skeletal disorders. Emerging evidence links ECM composition changes to muscle fibrosis and atrophy, highlighting mechanisms that impair regeneration [19]. Furthermore, novel insights into ECM involvement in autoimmune and inflammatory MSK diseases reinforce its role as both a pathological driver and therapeutic target [20]. The presented discoveries underscore the multifaceted functions of the ECM and its promise for guiding next-generation treatments in skeletal and muscle diseases.

## 3 Development of a novel cell-based gene therapy for intervertebral disc degeneration

Low back pain is the leading global cause of disability and a major MSK challenge [21]. IVD degeneration, involving breakdown of structural components, immunometabolic alterations, inflammation, pain, and impaired function, is a key contributor to Low back pain [22,23]. With aging populations and rising chronic conditions like obesity and diabetes, innovative treatments targeting IVD degeneration are urgently needed [24]. Current therapies primarily address symptoms without altering disease progression [25].

The Mobasheri lab focuses on the molecular and cellular basis of IVD degeneration, with the long-term goal of developing a novel cell-based gene therapy. Central to this is the cartilaginous endplate (CEP), a thin hyaline-like cartilage crucial for nutrient exchange between the disc vertebrae [26,27]. Despite its importance, the CEP remains understudied. Degeneration and calcification of the CEP compromise disc health; restoring its function may reverse IVD degeneration.

Cell and gene therapies targeting the root biological mechanisms such as inflammation, ECM breakdown, and nutrient deficiency hold promise. The lab's strategy involves overexpressing growth differentiation factor 6 (GDF6), a member of the transforming growth factor (TGF)-β superfamily critical for spinal development [28,29]. Mutations in GDF6 are associated with spinal deformities, and GDF6 promotes differentiation of adipose-derived mesenchymal stem cells into nucleus pulposus-like cells. Importantly, GDF6 receptor expression persists in degenerated disks, suggesting therapeutic potential [30,31].

Using recombinant adeno-associated virus vectors and CRISPR activation tools, the Mobasheri lab aims to enhance GDF6 expression to reprogram IVD and CEP cells. Initial experiments have focused on overexpressing GDF6 in HEK-293T cells, analysing gene expression via qPCR, and testing effects on inflammation and ECM synthesis. Parallel work develops therapeutic cells for *ex vivo* and *in vivo* degeneration models. The goal is to reduce inflammation, promote collagen type II synthesis, and restore CEP function, mitigating IVD degeneration [32].

## 4 Spatio-temporal modelling of osteoarthritis

The osteochondral unit [33] undergoes characteristic structural and functional alterations in OA [34]. When selecting appropriate animal species for OA studies, not only joint size but also the relative thickness of its components must be considered. Remarkably, humans, despite their large tibial plateau, possess a thin subchondral bone plate (similar to rabbits), and a thicker cartilage than any commonly used animal models [35]. These facts also result in the exceptionally high articular cartilage-to-bone plate thickness ratio (more than 2-fold higher than rabbits) [35].

Following OA induction, changes of articular cartilage gene expression appear already after 6 h [36], followed by synovial cell activation, mononuclear cell infiltration, and inflammation [37]. After 1 week, ECM degradation, including loss of lubricin [38], type II collagen and proteoglycans [39] begins. MRI studies have revealed a spatially heterogeneous cartilage loss, signifying the value of spatially resolved analyses [40]. Arthroscopic grading of human knees affected by early OA demonstrated a strong topographic correlation between meniscal and cartilage lesions [41]. To further investigate this, the Madry lab induced traumatic medial meniscus injuries in adult sheep. Over time, such localized meniscal damage led to similar spatially localized and expanding osteochondral OA changes, including cartilage damage, osteophyte formation, and subchondral bone thickening, faithfully modelling human OA progression [41].

Micro-CT imaging is increasingly utilized to study subchondral bone microstructure translational models. In mice, alterations are generally unidirectional, progressing toward sclerosis. Rats, however, exhibit a biphasic pattern: an early osteopenia and trabecular loss, followed by late-stage sclerosis [42]. In large animals with early to mid-stage OA, the subchondral bone plate becomes thicker and more porous, while trabecular structure diminishes [43].

Large animal models realistically replicate the spatial and temporal patterns of osteochondral changes seen in human knee OA. Careful attention to study design, laterality, and appropriate selection of controls is needed as they considerably impact translational study outcomes. Knowledge gaps exist especially for modelling late OA, which remains an important future research direction.

## 5 Osteoarthritis and chronic stress: Which came first?

Chronic stress is omnipresent in our modern 21st-century society and is regarded by the World Health Organization as one of the greatest contemporary health risks [44]. Chronic stress, induced by long-lasting and repeated or additive stimuli, activates the sympathetic nervous system and the hypothalamus–pituitary–adrenal axis, elevating norepinephrine and cortisol levels [45]. Increasing evidence shows that, besides affecting cardiovascular, metabolic, and mental health, chronic stress can also accelerate degenerative joint disorders such as OA [46]. Norepinephrine and cortisol have been shown to impair progenitor cell-driven cartilage regeneration [[47], [48], [49]], promote catabolic and pro-inflammatory pathways in cartilage [50,51] and subchondral bone [52], and, in synovial tissue, exert either pro- or anti-inflammatory effects, depending on the concentration, the duration of exposure, and receptor expression [53]. However, most studies focused only on single cell types, often neglecting the interactions between various joint tissues.

The Jenei-Lanzl group recently demonstrated in a mouse model of OA that chronic stress in fact exacerbates cartilage degeneration, synovial inflammation, and joint pain. Interestingly, experimental OA itself was shown to raise levels of norepinephrine and corticosterone, suggesting chronic stress might be both ‘the chicken and the egg’ in OA [54]. The logical hypothesis resulting from these findings was that late-stage knee OA patients had significantly lower heart rate variability and higher cortisol levels, indicating greater sympathetic tone and autonomic dysfunction [55]. Enhancing parasympathetic activity, for example by vagus nerve stimulation [56], might represent a potential treatment option for knee OA patients, although standardized approaches are still needed to ensure effective implementation. The presented studies underline the significance of systemic factors in OA pathogenesis and progression and support viewing this condition as a disease affecting the whole individuum [57].

## 6 Summary of the short talks from the University of Debrecen

### 6.1 Autoimmunity and immunotherapy in musculoskeletal diseases

The majority of musculoskeletal diseases involve complex immune dysregulation, with either or both autoinflammatory and autoimmune mechanisms contributing to tissue damage. Key drivers include cytokines like interleukin (IL)-1β and IL-18, B cell–mediated autoantibody production, and innate immune activation through inflammasomes [58]. In various autoimmune myopathies, CD19-CAR T cell therapy has shown promise by depleting pathogenic B cells, resetting the immune system and inducing sustained, drug-free clinical remission [59].

#### 6.1.1 The wide spectrum of autoimmunity and autoinflammation

Inflammatory rheumatic diseases and non-rheumatic conditions studied by the Szekanecz group range along the autoinflammatory-autoimmune spectrum. Autoinflammation primarily involves innate immunity, including macrophages, neutrophils, mast cells, and the complement system, while autoimmunity is driven by adaptive immunity with T, B cells and autoantibodies [60].

Autoinflammatory diseases include inflammasomopathies, relopathies (NFκB-related disorders), type I interferon-opathies, and conditions linked to endoplasmic reticulum stress [61,62]. The classical example is gout, driven by NLRP3 inflammasome activation. Here, NLRP3, leading to IL-1β and IL-18 secretion, is supported by gasdermin D [63]. IL-1β is a key cytokine in autoinflammation [64], driving inflammation, atherosclerosis, cartilage and bone destruction in inflammatory diseases [65]. The biomarkers of autoinflammation include CRP, S100 proteins (calprotectin), IL-18, and serum amyloid A [66].

Numerous MSK disorders exhibit autoimmune and autoinflammatory features, categorized as immune-mediated inflammatory diseases (IMID) or mixed-pattern diseases. Mixed-pattern diseases include RA, psoriasis, psoriatic arthritis, axial spondyloarthritis, inflammatory bowel disease, and uveitis, displaying a wide phenotypic spectrum [60]. For example, RA, traditionally classified as seropositive, can have 10–12 phenotypes with autoinflammatory traits [67].

Beyond traditional therapies based on diagnosis, pathogenesis-driven treatment strategies are emerging, targeting autoimmune, autoinflammatory, and mixed mechanisms. For example, autoimmune conditions often require azathioprine, leflunomide, or T/B cell blockade, while autoinflammation can be managed with colchicine or cytokine (IL-1β, IL-18, IL-36) inhibitors [67].

#### 6.1.2 Harnessing chimeric antigen receptor T cells for autoimmune musculoskeletal diseases

CAR-T cell therapy, involving genetic reprogramming of patient T lymphocytes with chimeric antigen receptors to target specific cells, such as CD19-expressing B cells [68], is a central focus of the Szoor lab. Since B cells are central to many autoimmune conditions through autoantibody production and inflammation, CD19 is an ideal target. Unlike rituximab (anti-CD20), which spares tissue-resident and memory B cells, CD19-CAR T cells deplete both peripheral and tissue B cells, a critical feature in diseases such as RA, systemic lupus erythematosus, and systemic sclerosis [69].

Clinical data show that CD19-CAR T cell therapy can induce drug-free remission, normalize cytokine profiles, and restore a naive B cell repertoire after depletion [69]. For example, a patient with refractory myositis showed complete remission, with improved muscle strength, lung function, and normalized autoantibodies and cytokines, using CD19-CAR T cells alongside mycophenolate mofetil to suppress CD8^+^ T cell–driven pathology [70]. Similarly, juvenile dermatomyositis patients achieved rapid B cell depletion and over eight months of symptom remission [71]. A study of 30 patients, including those with idiopathic inflammatory myopathies, showed significant clinical response and no disease flare-ups over a year [72].

Emerging evidence suggests that CD19-CAR T cells may revolutionize autoimmune myopathies, offering efficacy, immune modulation, and potential reversals of organ damage. As more evidence accumulates, these therapies could redefine the standards for refractory autoimmune diseases.

### 6.2 Muscle regeneration, a neglected player in the context of joint diseases

Skeletal muscle regeneration involves coordinated cytoskeletal remodelling, mitochondrial reprogramming, and immune regulation, with Septin7 as a crucial cytoskeletal protein required for muscle integrity and repair [73]. Its deficiency leads to disrupted ECM deposition and sustained inflammation, highlighting its dual role as a structural scaffold and regulator of regenerative processes. Complementarily, adenosine receptor signalling, such as the A3Rs and A2BRs, revealed as a powerful modulator of the inflammatory phase of muscle regeneration [74], suggesting that targeting cytoskeletal components and receptors together may offer synergistic strategies for enhancing muscle repair.

#### 6.2.1 The role of the cytoskeleton in muscle regeneration

The cytoskeleton is a dynamic, multifunctional structural network essential to many cellular processes [75]. Alongside the classical cytoskeletal components, i.e., actin filaments, microtubules, and intermediate filaments, the septin family is increasingly recognized as a fourth cytoskeletal element [73,76].

Septins are a conserved family of GTP-binding proteins. Humans express 13 septin isoforms that assemble into hetero-oligomeric complexes [77], which polymerize into filaments [78] and organize into higher-order structures, such as rings, bundles, and cage-like assemblies. Septin7 is essential for filament formation and structural integrity of these complexes [79].

Skeletal muscle regeneration restores muscle architecture and function following injury, mainly driven by satellite cells, quiescent muscle stem cells activated upon damage [80]. This process involves tightly regulated cytoskeletal remodelling and mitochondrial dynamics [81,82].

Studies from the Csernoch lab [73,82] confirm septin expression in murine skeletal muscle at transcript and protein levels. Septin7-deficient mice revealed severe musculoskeletal abnormalities, including kyphosis and weakened muscle force [82]. Ultrastructural analysis demonstrated disrupted mitochondrial organization and reduced function, indicating a role for Septin7 in mitochondrial integrity. In a barium chloride (BaCl_2_)-induced acute muscle injury model, Septin7 expression increased during regeneration, whereas Septin7 deficiency delayed regeneration, characterized by impaired myogenin expression kinetics, altered ECM deposition, and persistent inflammation.

The presented findings establish Septin7 as a critical regulator of skeletal muscle homeostasis and regeneration, functioning as both structural scaffold and modulator, highlighting its therapeutic potential for enhancing muscle repair.

#### 6.2.2 Involvement of adenosine receptors in the skeletal muscle repair following injury

Increasing evidence shows that inflammatory responses critically link initial muscle injury to subsequent regeneration following injury [83]. Adenosine, an endogenous anti-inflammatory nucleoside generated in high amounts during tissue damage [84], exerts its effects via four adenosine receptor types [85]. Among these, adenosine A3 receptors (A3Rs) are absent from the skeletal muscle but expressed on various inflammatory cells, while adenosine A2A (A2ARs) and A2B (A2BRs) receptors are expressed by both muscle and inflammatory cells [86,87].

The Szondy lab investigated the effects of deleting these ARs on tibialis anterior muscle regeneration post-cardiotoxin injury in mice. They found A3Rs and A2BRs exert reciprocal regulation on initial inflammation and transmigration of inflammatory cells: A2Rs promote, while A3Rs inhibit these processes. Consequently, muscles lacking A3Rs exhibited faster debris clearance, accelerated satellite cell proliferation and differentiation, and increased myoblasts fusion, leading to more rapid muscle regeneration and larger myofibers [88]. Conversely, A2AR deficiency impaired myoblast fusion and hypertrophy, resulting in smaller myofibers and reduced muscle mass. The presented data indicate therapeutic potential in inhibiting A3Rs or activating A2BRs to enhance skeletal muscle regeneration following injury.

### 6.3 Role of stem cell differentiation in diagnostic and therapeutic perspectives of musculoskeletal disorders

Elevated expression of the Runt-related transcription factor RUNX3 in mouse embryonic stem cells enhances early mesodermal differentiation by upregulating collagen gene expression, which may influence both cell adhesion and lineage specification [89]. Since ECM composition and mechanical cues are key regulators of MSK development, the ability of cyclic mechanical loading to entrain circadian rhythms in chondrogenic cells highlights a critical interplay between biomechanical and transcriptional regulation [90]. Together, these findings suggest that modulating both intrinsic genetic programs (e.g., via RUNX3) and extrinsic mechanical signals could synergistically optimize MSK tissue engineering.

#### 6.3.1 Mesodermal differentiation via RUNX3 modulation

Forced expression of gene regulatory proteins is a robust tool to modulate cell fate. The RUNX family of transcription factors, comprising RUNX1, RUNX2, and RUNX3, modulate critical processes in cellular development, including proliferation and lineage specification [91]. In mice, *Runx1* knockout abolishes definitive haematopoiesis, *Runx2* deficiency impairs bone formation, and *Runx3* loss leads to limb ataxia due to neuronal death during embryogenesis. RUNX3 is also involved in haematopoietic development [92].

The Szatmári lab investigates the impact of RUNX3 on ESC-derived mesodermal and haematopoietic progenitors. Recent RNA sequencing of RUNX3-primed mouse embryonic stem cells identified over 900 differently expressed genes upon transgenic RUNX3 induction [93], including upregulation of mesoderm differentiation genes (*Hand1*, *Kdr,* and *Sox7*) and many collagen-coding genes (*Col1a2, Col4a5, Col5a1, Col6a1, Col7a1, Col8a1, Col8a2, Col11a1,* and *Col12a1*). They also investigated the effects of RUNX3 during early mesodermal specification: in 8-day and 12-day differentiated ESCs, RUNX3 increased expression of several collagen transcripts (e.g., *Col1a1*, *Col7a1, Col8a1, Col9a2, Col11a1, Col11a2, Col23a1* and *Col26a1*). Together, the presented data suggest that RUNX3 coordinates collagen synthesis and significantly influences early mesodermal development in mouse ESC-differentiated cells.

#### 6.3.2 Mechanical loading entrains circadian rhythms in chondrogenic cells – a transcriptomic analysis

Circadian rhythms regulate key physiological processes in musculoskeletal tissues, including cartilage homeostasis, and are a major focus of the Matta lab. Mechanical stimuli can serve as “*Zeitgeber*” (time-giver) for peripheral clocks in these tissues [90]. The presented study examined the impact of cyclic uniaxial mechanical loading on gene expression during early *in vitro* chondrogenesis, comparing it to serum shock, a conventional circadian clock entrainment method.

Chicken limb bud-derived chondroprogenitor micromass cultures were exposed to either 60 min of uniaxial dynamic loading or serum shock on culture day 1. Samples for RNA sequencing were collected every 6 h from 24 to 72 h post-stimulation. Principal component analysis revealed distinct transcriptomic changes induced by mechanical loading, segregating these cultures from both controls and serum-shocked groups. Pathway analysis confirmed circadian rhythm alterations.

These findings suggest that mechanical loading effectively entrains the molecular clockwork in chondrogenic cells, producing coordinated gene expression patterns. Understanding how mechanical stimuli interact with circadian clocks may enhance cartilage tissue engineering and regenerative strategies by optimizing mechanical stimulation protocols to synchronize intrinsic circadian rhythms and improve functional properties of engineered cartilage.

## 7 Concluding remarks

The third UD-MUSK has solidified its status as a pivotal platform for advancing MSK research in Central and Eastern Europe. By convening leading experts and early-career researchers from across Europe, the symposium fostered robust interdisciplinary collaboration, catalysing the exchange of innovative ideas and new research partnerships.

This year's symposium covered critical areas including IVD degeneration, OA, and broader MSK disorders. Emphasis was placed on immunological mechanisms, the bidirectional influence of chronic stress interactions and tissue degeneration, and the crucial yet often overlooked role of muscle regeneration in joint pathologies. The integration of molecular, cellular, and translational research, from novel gene therapies targeting IVD degeneration to the dual role of ECM fragments as biomarkers and therapeutic agents, highlighted the necessity to integrate fundamental research with clinical perspectives to address the multifactorial complexity of MSK conditions.

Importantly, the symposium revealed emerging evidence that MSK tissue deterioration may be reversible, provided that diagnostic and therapeutic efforts target early molecular and cellular events. The complex relationship between chronic stress and joint degeneration supports a holistic, system-level approach to research and care. The focus on muscle biology, often overlooked at global meetings, provided a more comprehensive understanding of MSK health and pathology.

The symposium also expanded its scope by explicitly addressing the fundamental challenges of translating advanced biomarkers, therapeutic strategies, and disease modelling into clinical realities. These challenges encompass scientific hurdles in validation and reproducibility, regulatory complexities, and practical considerations in implementation. By emphasizing these barriers, the symposium underscored the critical need for coordinated efforts bridging discovery and clinical application, as highlighted by several presenters.

The sustained increase in attendance and engagement, particularly from Hungary and neighbouring regions, attests to the unique value of the symposium in an underserved area. The University of Debrecen's continued commitment to this annual event promises to further elevate the international profile of Central and Eastern European MSK research, while expanding the global scientific network devoted to reducing the burden of OA and related disorders.

Key challenges and future directions presented at the symposium are summarized in Table 1. These include complex ECM roles in disease and regeneration; the need for CEP-targeted therapies in IVD degeneration; systemic factors like chronic stress in OA progression; refined immune-pathogenesis-based treatments for autoimmune and autoinflammatory disorders; the promise CAR-T cell therapy for refractory autoimmune myopathies; and the critical regulatory functions of cytoskeletal, receptor-mediated, and transcriptional mechanisms in muscle and cartilage repair. Insights into mechanical entrainment of circadian rhythms also suggest novel approaches for optimizing cartilage tissue engineering.

**Table 1:** A summary of the key challenges musculoskeletal research is currently facing, as identified by each speaker of the symposium, listed as key points from each section of the manuscript, alongside suggested future research directions aimed at addressing these challenges. The compilation highlights critical knowledge gaps, emerging therapeutic approaches, and innovative strategies across diverse aspects of musculoskeletal biology and disease.

Manuscript Section	Key Messages and Current Challenges in Musculoskeletal Research	Suggested Future Directions
Extracellular matrix in skeletal diseases	Complex ECM composition and degradation in diseases like OA; identifying function of fragments with dual catabolic vs. regenerative roles	Systematic ECM degradation analysis to find novel biomarkers and pro-regenerative therapeutic targets
Novel cell-based gene therapy for IVD degeneration	Ineffective current therapies targeting only symptoms, insufficient CEP research in IVD degeneration	Develop CEP-targeted gene/cell therapies enhancing GDF6 expression to restore disc function and reduce inflammation
Spatio-temporal modelling of osteoarthritis	Large animal models effectively mimic human knee OA patterns but study design impacts outcomes; knowledge gaps exist in modelling late-stage OA	Improve study designs with attention to laterality and controls; focus research on late-stage OA modelling
Osteoarthritis and chronic stress	Bidirectional influence of chronic stress hormones on OA progression; systemic factors often overlooked	Target autonomic dysfunction (parasympathetic strengthening) as adjunct therapy; holistic view of OA as whole-person disease
Autoimmunity and autoinflammation	Overlapping immune-mediated inflammatory diseases complicate diagnosis and therapy	Pathogenesis-based personalized therapies targeting both autoimmune and autoinflammatory pathways
CAR T cells for autoimmune MSK diseases	Conventional therapies insufficient for refractory autoimmune myopathies; need deep immune modulation	Use CD19-CAR T cells to achieve durable remission and immune reconstitution; expand *in vivo* CAR T approaches
Cytoskeleton in muscle regeneration	Limited understanding of cytoskeletal elements such as Septin7 in muscle regeneration and mitochondrial function	Target Septin7 to improve muscle repair and regeneration through modulation of cytoskeletal scaffolding and mitochondrial integrity
Adenosine receptors in muscle repair	Complex receptor-mediated regulation of inflammatory response and muscle regeneration	Therapeutically modulate A3/A2B receptors to optimize inflammation resolution and enhance muscle repair
RUNX3 in mesodermal differentiation	Controlling differentiation and ECM production in ESC-derived progenitors remains challenging	Harness RUNX3 to coordinate collagen synthesis and guide early mesodermal differentiation for regenerative applications
Mechanical loading and circadian rhythms in chondrogenesis	Mechanisms by which mechanical signals entrain circadian rhythms in cartilage formation are unclear	Utilize mechanical stimulation to entrain circadian clocks improving cartilage engineering and functional integration

The organizers encourage ongoing and broader involvement from all MSK researchers. Strong collaboration is fundamental to driving breakthroughs and improving patient outcomes. By sharing novel discoveries, resources, and fostering interdisciplinary partnerships, UD-MUSK continues to push the frontiers of MSK research toward more precise diagnostics, effective regenerative therapies, and holistic management strategies for OA, RA, and associated disorders.

## CRediT author statement

CM conceived the idea of the workshop and worked with to develop the agenda and workshop. All authors (organizing group members and invited speakers) reviewed the minutes from the workshop and contributed to and reviewed the manuscript prior to submission. All authors made substantial contributions to writing of the original outline, discussion of the content, and reviewing/editing of the article before submission. All authors approved the final version for publication and agree to be accountable for the accuracy and integrity of the study.

**Patrik Kovács:** Writing - Original Draft; Writing - Review & Editing.

**Judit Vágó:** Writing - Original Draft; Writing - Review & Editing.

**Ali Mobasheri:** Conceptualization; Writing - Original Draft; Writing - Review & Editing; Project administration; Funding acquisition.

**Zsuzsa Jenei-Lanzl:** Writing - Original Draft; Writing - Review & Editing; Project administration; Funding acquisition.

**Frank Zaucke:** Writing - Original Draft; Writing - Review & Editing; Project administration; Funding acquisition.

**Henning Madry:** Writing - Original Draft; Writing - Review & Editing; Project administration; Funding acquisition.

**László Csernoch:** Writing - Original Draft; Writing - Review & Editing; Project administration; Funding acquisition.

**Zoltán Szekanecz:** Writing - Original Draft; Writing - Review & Editing; Project administration; Funding acquisition.

**Zsuzsa Szondy:** Writing - Original Draft; Writing - Review & Editing; Project administration; Funding acquisition.

**Árpád Szöőr:** Writing - Original Draft; Writing - Review & Editing; Project administration; Funding acquisition.

**Istvan Szatmari:** Writing - Original Draft; Writing - Review & Editing; Project administration; Funding acquisition.

**Tamás Oláh:** Conceptualization; Writing - Original Draft; Writing - Review & Editing; Project administration; Funding acquisition.

**Csaba Matta:** Conceptualization; Writing - Original Draft; Writing - Review & Editing; Project administration; Funding acquisition.

## Role of the funding source

Funding bodies were not involved in the study design, data collection, analysis, or interpretation, or in the writing of the manuscript. The decision to submit this paper for publication was not influenced by any funding bodies.

AM wishes to acknowledge the generous financial support of the Research Council of Lithuania/Lietuvos Mokslo Taryba, Research Council of Finland/Suomen Akatemia Profi 6, grant numbers 336449 and 351568, awarded to the University of Oulu. AM also acknowledges funding from Horizon Europe (PROTO-Advanced PeRsOnalized Therapies for Osteoarthritis-TACKLING INFLAMMATION TO IMPROVE PATIENT OUTCOMES, grant agreement ID 101095635, https://cordis.europa.eu/project/id/101095635 and ENgineered CArtilage from Nose for the Treatment of Osteoarthritis (ENCANTO), grant agreement ID 101137315, https://cordis.europa.eu/project/id/101137315). This paper was supported by the János Bolyai Research Scholarship of the Hungarian Academy of Sciences, awarded to CM and TO. CM and AS were supported by the Young Researcher Excellence Program (grant numbers: FK-134304 and 152166, as well as FK-132773, respectively) of the National Research, Development and Innovation Office, Hungary. LC and Z. Szondy acknowledge the funding from the Hungarian National Research, Development and Innovation Office, project no. K-137600 and K-138162, respectively. This research was supported by the EKÖP-24-2 University Research Fellowship Program of the Ministry for Culture and Innovation from the source of the National Research, Development and Innovation Fund, Hungary, awarded to PK (EKÖP-24-3-II-DE-1). CM and AM also acknowledge financial support from the European Cooperation in Science and Technology COST Association Action CA21110–Building an open European Network on OsteoArthritis research (NetwOArk). This paper was supported by the University of Debrecen Program for Scientific Publication. ZJL and FZ were supported by the Stiftung Friedrichsheim, Frankfurt, Germany. ZJL and FZ were supported by the German Research Foundation (Deutsche Forschungsgemeinschaft; DFG) with FOR 2407 (JE 642/4-2 to ZJL (FOR 2407 project number 277277765)) and FOR 2722 (ZA 561/3-2 to FZ (FOR2722 project number 384170921). HM was supported by a grant from the DFG; funding number: DFG MA 2363/6-1, project number 517789576. This research was supported by the European Union and the State of Hungary and co-financed by the European Social Fund in the framework of TAMOP-4.2.4.A/2–11/1-2012-0001 ‘National Excellence Program’ (Z. Szekanecz), and by the European Union grant GINOP-2.3.2-15-2016-00050 (Z. Szekanecz).

## Declaration of competing interest

The authors declare that they have no conflicts of interest. The authors wrote this paper within the scope of their academic and research positions. None of the authors has any relationships that could be construed as biased or inappropriate.
